# Pretreatment HIV Drug Resistance and the Molecular Transmission Network Among HIV-Positive Individuals in China in 2022: Multicenter Observational Study

**DOI:** 10.2196/50894

**Published:** 2023-11-17

**Authors:** Hongli Chen, Jingjing Hao, Jing Hu, Chang Song, Yesheng Zhou, Miaomiao Li, Jin Chen, Xiu Liu, Dong Wang, Xiaoshan Xu, Peixian Xin, Jiaxin Zhang, Lingjie Liao, Yi Feng, Dan Li, Stephen W Pan, Yiming Shao, Yuhua Ruan, Hui Xing

**Affiliations:** 1 National Center for AIDS/STD Control and Prevention (NCAIDS) Chinese Center for Disease Control and Prevention (China CDC) Beijing China; 2 Sichuan Nursing Vocational College Chengdu China; 3 Department of Public Health The University of Texas at San Antonio San Antonio, TX United States

**Keywords:** HIV, human immunodeficiency virus, mutation, pretreatment drug resistance, risk factors, molecular transmission network

## Abstract

**Background:**

Emerging HIV drug resistance caused by increased usage of antiretroviral drugs (ARV) could jeopardize the success of standardized HIV management protocols in resource-limited settings.

**Objective:**

We aimed to characterize pretreatment HIV drug resistance (PDR) among HIV-positive individuals and risk factors in China in 2022.

**Methods:**

This cross-sectional study was conducted using 2-stage systematic sampling according to the World Health Organization’s surveillance guidelines in 8 provincial-level administrative divisions in 2022. Demographic information and plasma samples were obtained from study participants. PDR was analyzed using the Stanford HIV drug resistance database, and the Tamura-Nei 93 model in HIV-TRACE was used to calculate pairwise matches with a genetic distance of 0.01 substitutions per site. Logistic regression was used to identify and estimate factors associated with PDR.

**Results:**

PDR testing was conducted on 2568 participants in 2022. Of the participants, 34.8% (n=893) were aged 30-49 years, 81.4% (n=2091) were male, and 3.2% (n=81) had prior ARV exposure. The prevalence of PDR to protease and reverse transcriptase regions, nonnucleoside reverse transcriptase inhibitors, nucleoside reverse transcriptase inhibitors, and protease inhibitors were 7.4% (n=190), 6.3% (n=163), 1.2% (n=32), and 0.2% (n=5), respectively. Yunnan, Jilin, and Zhejiang had much higher PDR incidence than did Sichuan. The prevalence of nonnucleoside reverse transcriptase inhibitor–related drug resistance was 6.1% (n=157) for efavirenz and 6.3% (n=163) for nevirapine. Multivariable logistic regression models indicated that participants who had prior ARV exposure (odds ratio [OR] 7.45, 95% CI 4.50-12.34) and the CRF55_01B HIV subtype (OR 2.61, 95% CI 1.41-4.83) were significantly associated with PDR. Among 618 (24.2%) sequences (nodes) associated with 253 molecular transmission clusters (size range 2-13), drug resistance mutation sites included K103, E138, V179, P225, V106, V108, L210, T215, P225, K238, and A98.

**Conclusions:**

The overall prevalence of PDR in China in 2022 was modest. Targeted genotypic PDR testing and medication compliance interventions must be urgently expanded to address PDR among newly diagnosed people living with HIV in China.

## Introduction

With the implementation of the “Treat all” program in 2016 in China, all people living with HIV are eligible to be treated, with their consent, regardless of their CD4 cell count and viral load level. Early treatment has been shown to effectively prolong the life span of people living with HIV and prevent secondary transmission. However, treatment of all individuals diagnosed with HIV has the potential to increase the prevalence of HIV drug resistance (DR) [1-4]. Pretreatment HIV drug resistance (PDR) refers to resistance that is detected among antiretroviral drug (ARV)–naive people initiating antiretroviral therapy (ART) or people with prior ARV drug exposure [5]. PDR may compromise the effectiveness of ART, increase mortality, and lead to secondary transmission of HIV [6]. Thus, assessing PDR levels is important for informing national policies on ARV and for improving ART programs and service delivery.

In China, individual ART plans are determined primarily by national standards for first- and second-line regimens, and potential DR is not a major consideration among individuals preparing to initiate ART. Due partly to resource limitations, individual HIV genotype resistance testing is not routinely conducted prior to the initiation of ART in China. This approach to developing individual ART regimens may be associated with DR [7]. A national meta-analysis indicated that PDR rapidly rose from 4.05% in 2011 to 5% in 2017. Specifically, resistance to nonnucleoside reverse transcriptase inhibitors (NNRTIs) increased from 2.15% to 3.81% from 2012 to 2017 [8]. However, it is important to note that PDR testing and prevalence varies widely by geographic region. Other surveys conducted on PDR in certain provinces of China showed that the prevalence of PDR was 10.5% in Chongqing, 9.1% in Shenyang, 4.1% in Beijing, 6% in Shenzhen, and 10.8% in Pu’er County of Yunnan [1,9-12]. The rates of PDR among HIV-positive individuals in China are comparatively lower than those in other countries, such as Cameroon (10.4%), Namibia (12.7%), Israel (12.1%), Mozambique (14.4%), Mexico (14.8%), the United States (22.5%), and Japan (12.5%) [13-19]. Yet China still lacks PDR surveillance methods that meet World Health Organization (WHO) standards. Assessing PDR prevalence with WHO standardized protocols will facilitate more accurate estimates of PDR prevalence in China, which will in turn better inform evidence-based ART policies in China.

Molecular transmission network analysis can be used to identify the maximum number of clusters and relatively active clusters based on genetic distance thresholds. Such methods have been used to evaluate the efficacy of ART on preventing secondary HIV transmission [20]. Moreover, our understanding of DR has been greatly enhanced by studies integrating analysis of transmission characteristics, DR strains, and molecular transmission networks [10,21].

The WHO has identified DR strategic surveillance of PDR as a very high priority in countries using efavirenz (EFV)/nevirapine (NVP) for first-line ART and recommends implementation or updates every 3 years [22]. Based on WHO PDR surveillance guidelines, the aim of this novel multicenter observational study is to investigate the prevalence of PDR and drug resistance mutations (DRMs) to partial pol regions, including protease (PR) and reverse transcriptase regions (RT), in diverse provinces in China. 

## Methods

### Study Design and Study Population

We selected 8 provincial-level administrative divisions (PLADs) in China as the study area to conduct a large multicenter observational cross-sectional study. Two-stage systematic sampling in each PLAD was conducted according to the WHO PDR surveillance guidelines [23]. Provinces were stratified into the following 3 categories based on their HIV incidence rates: high (>2.00 cases per 10,000 individuals; included Sichuan Province, Chongqing Municipality, and Yunnan Province), moderate (0.52-1.99 cases per 10,000 individuals; included Jiangsu Province and Zhejiang Province), and low (<0.52 cases per 10,000 individuals; included Jilin Province, Hebei Province, and Hubei Province).

Guided by WHO PDR surveillance guidelines, we calculated the minimum necessary sample size by assuming the prevalence of PDR was 10% and the CI range was ±5%. We sampled 11-20 clinics in each PLAD according to the number of HIV-positive individuals starting or restarting ART in the past 12 months. The minimum sample size in each PLAD was 158-179 samples. To ensure a minimum necessary sample size, we assumed that 40% of collected specimens would be lost to factors such as laboratory failure and a design effect. Expected and actual sample sizes for each province are shown in Multimedia Appendix 1. Recruitment and specimen collection of HIV-positive individuals occurred in 2022 as individuals initiated ART.

The study eligibility criteria for individuals included the following: (1) tested HIV-positive, (2) ≥18 years old, (3) started or restarted first-line ART between January and June 2022 (includes women exposed to ARV drugs for preventing mother-to-child transmission of HIV, people who have received pre-exposure prophylaxis, and individuals reinitiating first-line ART), and (4) provided informed consent.

### Data Collection

Each study participant was assigned a confidential and unique patient identifier used for linking basic epidemiological information and specimens. Basic sociodemographic data were collected, including age, sex, ethnicity, education level, marital status, occupation, route of infection, prior ARV exposure, time elapsed since HIV diagnosis, and PLADs. Clinical information included patients’ CD4 cell count before ART and HIV subtype.

### Laboratory Tests

Viral RNA was extracted from 200 µL plasma samples using the QIAamp viral RNA mini kit (Qiagen, Hilton, Germany) according to the operational instructions. Extracted RNA was used for first-round polymerase chain reaction, and the products were used for second-round polymerase chain reaction amplification; both rounds were performed in 25 µL volume reactions to amplify the HIV pol gene region (HXB2: 2253-3553 nt). This covered the PR region (4-99 amino acids) and the partial RT region (1-251 amino acids) [24]. The amplified products were sequenced by Sanger sequencing.

### Subtype and Drug Resistance Analysis

Sequencher (version 4.10.1, Gene Codes Corp) software was used to recombine and edit the original sequence fragments and nucleosides. BioEdit (version 7.0.9, Informer Technologies) software was used to realign the HXB2 reference sequence. The sequences were realigned, and some common reference sequences (including A to K) were used to identify the subtype by constructing neighbor joining phylogenetic trees in MEGA (version 7.0.26, MEGA Software). Subtypes with a bootstrap value higher than 70% were determined using HXB2 as the reference sequence. Sequences were realigned using the HIV database website [25]. Realigned sequences were uploaded to the Stanford Drug Resistance Database [26] to identify the degree of resistance of the 12 listed drugs that the WHO recommends for monitoring, namely, EFV, NVP, abacavir, zidovudine, lamivudine, tenofovir, emtricitabine, stavudine, didanosine, lopinavir/r, atazanavir/r, and darunavir/r. Corresponding DR mutant genes with levels of 1, 2, 3, 4, and 5 were classified as sensitive, potential resistance, low resistance, moderate resistance, and high resistance, respectively. Only mutations characterized as low, moderate, or high resistance were considered resistant. All possible sample mutual contamination, laboratory contamination, and other sequence quality controls were monitored by constructing neighbor joining phylogenetic trees and using the WHO HIVDR QC tool [27].

### Construction of the Molecular Network

Realigned sequences were conducted to calculate pairwise genetic distances though the Tamura-Nei 93 model in the transmission cluster engine, HIV-TRACE [28]. Sequences no longer than 1000 bp or containing ≥5% ambiguities were excluded. A threshold genetic distance of 0.01 substitutions per site was used to observe transmission events that may occur within 5-6 years [29]. Each individual in the molecular network was represented by a node, and each node in the molecular transmission network represented HIV-positive individuals who were matched with epidemiological information. We then linked nodes to each other if their pairwise genetic distance was up to 0.01 substitutions per site. Web pages were used to visualize molecular networks, which were based on technical guides for HIV transmission network monitoring and intervention [30]. The results and subsequent molecular network diagram are accessible on the web [31]. Our previous studies used other software, such as HYPHY to calculate the pairwise gene distance and Cytoscape for visualization [20,32,33]. Clusters were defined as containing 2 or more nodes with HIV-positive individuals in the same cluster having potential for transmission.

### Statistical Analysis

All quantitative data analyses were performed using the Statistical Analysis System version 9.4 (SAS Institute). Two-sided *P* values <.05 were considered statistically significant. All variables were assessed using univariate binary logistic regression. Variables significantly associated (*P*<.05) with the outcome were entered into a stepwise regression model to remove collinear variables. The remaining variables were entered into a multivariate binary logistic regression model to identify correlates of PDR. The *P* value of the final multivariate logistic regression model was <.05.

### Ethical Considerations

This study was approved by the ethics committee of the National Center for AIDS/STD Control and Prevention, China Centers for Disease Control and Prevention (approval number X140617334).

## Results

### Characteristics of the Study Participants

A total of 2869 HIV-positive individuals were initially recruited into the study. After removal of 16 HIV-positive individuals who were younger than 18 years and 9 individuals who had not started ART, the penultimate sample size was 2844. PDR test results were available for 90.3% (n=2568) of participants in RT/PR regions, thus yielding a final sample size of 2568. Of the 2568 study participants with valid PDR results, 34.8% (n=893) were aged 30-49 years, 81.4% (n=2091) were male, 86.8% (n=2229) were of Han ethnicity, 30.5% (n=783) had a junior high school education, 37% (n=950) were single, 36% (n=924) were farmers, and 57.6% (n=1480) and 37% (n=951) were infected through heterosexual and homosexual intercourse, respectively. In addition, 3.2% (n=81) of participants had prior ARV drug exposure, 84.5% (n=2170) were diagnosed in 2022, and 37.5% (n=962) had CD4 cell counts of 0-199 cells/mm^3^ prior to ART initiation (Table 1).

**Table 1:** Characteristics of HIV-positive individuals in 2022 in China.

Characteristic		Participants (n=2568), n (%)
**Age (years)**		
	18-29	606 (23.6)
	30-49	893 (34.8)
	50-69	894 (34.8)
	≥70	175 (6.8)
**Sex**		
	Male	2091 (81.4)
	Female	477 (18.6)
**Ethnicity**		
	Han	2229 (86.8)
	Other	339 (13.2)
**Education**		
	Primary and below	797 (31)
	Junior high school	783 (30.5)
	Senior high school	360 (14)
	College	607 (23.6)
	Missing	21 (0.9)
**Marital status**		
	Single	950 (37)
	Married or cohabiting	1144 (44.6)
	Divorced or widowed	457 (17.8)
	Missing	17 (0.6)
**Occupation**		
	Farmer	924 (36)
	Other	1644 (64)
**Route of infection**		
	Heterosexual intercourse	1480 (57.6)
	Homosexual intercourse	951 (37)
	Intravenous drug use	21 (0.8)
	Other	116 (4.5)
**Prior ARV^a^ exposure**		
	Yes	81 (3.2)
	No	2487 (96.8)
**CD4 cell count before ART^b^ (cell/mm^3^)**		
	0-199	962 (37.5)
	≥200	1554 (60.5)
	Missing	52 (2)

^a^ARV: antiretroviral drugs.

^b^ART: antiretroviral therapy.

### PDR and Mutations

Among the 2568 included study participants, PDR was detected in 7.4% (n=190). Table 2 shows the prevalence of overall PDR in Hebei (13/175; 7.4%), Jilin (19/206; 9.2%), Jiangsu (24/335; 7.2%), Zhejiang (34/385; 8.8%), Hubei (20/362; 5.5%), Chongqing (20/378; 5.3%), Sichuan (17/350; 4.9%) and Yunnan (43/377; 11.4%) in 2022. The prevalence of PDR in Yunnan (*P*=.002), Jilin (*P*=.047), and Zhejiang (*P*=.04) was much higher than that in Sichuan. Additionally, the prevalence of PDR in Yunnan was significantly higher than those in Chongqing (*P*=.002) and Hubei (*P*=.004).

Of the 2568 cases studied, the prevalence of PDR to NNRTIs, nucleoside reverse transcriptase inhibitors (NRTIs), and protease inhibitors (PIs) were 6.3% (n=163), 1.2% (n=32), and 0.2% (n=5), respectively. NNRTI-related DR to EFV and NVP was 6.1% (n=157) and 6.3% (n=163), respectively. There were 14 NNRTI-related resistance mutations; the most common mutations were K103N/S (n=81, 3.2%), followed by V179D/E/IL (n=39, 1.5%), and E138A/G/K (n=31, 1.2%). Among NRTI-related DR, the most frequent PDR drug was abacavir (n=19, 0.7%), followed by stavudine (n=17, 0.7%), emtricitabine (n=15, 0.6%), lamivudine (n=15, 0.6%), didanosine (n=13, 0.5%), zidovudine (n=10, 0.4%), and tenofovir (n=8, 0.3%). We detected 10 NRTI-related resistance mutations, of which M184V/I (n=13, 0.5%) was the most common. Even in the PR region, 0.2% (n=5) PI-related PDR to atazanavir/r and lopinavir/r was detected, including the PI-related DRMs L90M/IM (n=3, 0.1%), N88T/D (n=1, 0.1%), and V82IM (n=1, 0.1%) (Table 3).

**Table 2:** Prevalence of pretreatment HIV drug resistance by provincial-level administrative divisions in 2022 in China.

Province	Prevalence, n (%)					Participants (n=2568), n
	PR/RT^a^ (n=190)	NNRTIs^b^ (n=163)	NRTIs^c^ (n=32)	PIs^d^ (n=5)		
Hebei	13 (7.4)	9 (5.1)	4 (2.3)	0 (0)	175	
Jilin	19 (9.2)	17 (8.2)	4 (1.9)	0 (0)	206	
Jiangsu	24 (7.2)	22 (6.5)	1 (0.2)	1 (0.2)	335	
Zhejiang	34 (8.8)	27 (7)	7 (1.8)	0 (0)	385	
Hubei	20 (5.5)	19 (5.2)	1 (0.2)	0 (0)	362	
Chongqing	20 (5.3)	14 (3.7)	7 (1.8)	1 (0.2)	378	
Sichuan	17 (4.9)	16 (4.5)	0 (0)	1 (0.2)	350	
Yunnan	43 (11.4)	39 (10.3)	8 (2.1)	2 (0.5)	377	
All provinces	190 (7.4)	163 (6.3)	32 (1.2)	5 (0.2)	2568	

^a^PR/RT: protease and reverse transcriptase.

^b^NNRTIs: nonnucleoside reverse transcriptase inhibitors.

^c^NRTIs: nucleoside reverse transcriptase inhibitors.

^d^PIs: protease inhibitors.

**Table 3:** Pretreatment HIV drug resistance and mutations among HIV-positive individuals in 2022 in China.

Antiretroviral drug			Prevalence, n		% (95% CI)		HIV drug resistance mutations and combination of mutations, n (%)
PR/RT^a^ region (NNRTIs^b^, NRTIs^c^, PIs^d^)			190		7.4 (6.4-8.4)		
**NNRTIs**			163		6.3 (5.4-7.3)		
	Efavirenz	157		6.1 (5.2-7)		K103N/S: 81 (3.2)	
	Nevirapine	163		6.3 (5.4-7.3)		V179D/E/IL: 39 (1.5) V179D+E138A:14 (0.5) V179E+E138G: 11 (0.1) V179D+V106I: 2 (0.1) V179E+K238N: 2 (0.1) V179D+G190E: 1 (0.1) V179D+Y181C+G190A+H221Y: 1 (0.1) V179D+Y188F+K238T: 1 (0.1) V179E+A98G: 1 (0.1) V179E+K103N+E138A: 1 (0.1) V179E+K103N+E138G: 1 (0.1) V179E+K103N+V106M: 1 (0.1) V179E+V108I: 1 (0.1) V179E+H221Y: 1 (0.1) V179IL+Y188L: 1 (0.1) E138A/G/K: 31 (1.2) V106A/M/I: 15 (0.6) V106I+K103N: 5 (0.2) V106I+V179D: 2 (0.1) V106I+Y188L: 1 (0.1) V106I+G190A: 1 (0.1) G190C/A/E/S: 13 (0.5) K101E/P: 9 (0.4) Y181C: 9 (0.4) A98G: 8 (0.5) P225H: 8 (0.3) Y188F/L/C: 6 (0.2) V108I: 5 (0.2) H221Y: 5 (0.2) K238T: 3 (0.1) K238N+V179E: 2 (0.1) L100I/V: 2 (0.1)	
**NRTIs**			32		1.2 (0.8-1.7)		
	Abacavir	19		0.7 (0.4-1.1)		M184V/I: 13 (0.5)	
	Emtricitabine	15		0.6 (0.3-0.9)		T215A/S/I: 5 (0.2)	
	Lamivudine	15		0.6 (0.3-0.9)		T69D/DN/ADN/del: 5 (0.2)	
	Didanosine	13		0.5 (0.2-0.8)		D67N: 5 (0.2)	
	Stavudine	17		0.7 (0.3-1)		K70E/T/R: 5 (0.2)	
	Tenofovir	8		0.3 (0.1-0.5)		K65R: 3 (0.1)	
	Zidovudine	10		0.4 (0.1-0.6)		L210W/MRW: 2 (0.1) L74I: 2 (0.1) K219Q: 1 (0.1) Y115F: 1 (0.1)	
**PIs**			5		0.2 (0-0.3)		
	Atazanavir/r	4		0.1 (0-0.3)		L90M/IM: 3 (0.1)	
	Darunavir/r	0				N88T: 1 (0.1)	
	Lopinavir/r	4		0.1 (0-0.3)		V82IM: 1 (0.1)	
Mutual drug resistance to NNRTIs and NRTIs			10		0.4 (0.1-0.6)		

^a^PR/RT: protease and reverse transcriptase.

^b^NNRTIs: nonnucleoside reverse transcriptase inhibitors.

^c^NRTIs: nucleoside reverse transcriptase inhibitors.

^d^PIs: protease inhibitors.

### Factors Associated With PDR

Table 4 lists the factors associated with PDR in PR/RT regions. Compared with participants without prior ARV drug exposure, those who had prior ARV drug exposure had 7.45 times greater odds of PDR (odds ratio [OR] 7.45, 95% CI 4.50-12.34). Compared to participants with the CRF07_BC subtype, those with the CRF55_01B subtype had 2.61 times greater odds of PDR (OR 2.61, 95% CI 1.41-4.83).

**Table 4:** Factors associated with pretreatment HIV drug resistance (PDR) among HIV-positive individuals in 2022 in China.

Variable			Number (n=2568)		PDR (n=190), n (%)		Odds ratio (95% CI)		*P* value		Adjusted odds ratio (95% CI)		*P* value	
**Age (years)**														
	18-29	606		49 (8.1)		1.00		N/A^a^		—^b^		—		
	30-49	893		68 (7.6)		0.74 (0.64-1.37)		.74		—		—		
	50-69	894		60 (6.7)		0.82 (0.55-1.21)		.32		—		—		
	≥70	175		13 (7.4)		0.91 (0.48-1.72)		.78		—		—		
**Sex**														
	Male	2091		149 (7.1)		1.00		N/A		—		—		
	Female	477		41 (8.6)		1.23 (0.85-1.76)		.27		—		—		
**Ethnicity**														
	Han	2229		158 (7.1)		1.00		N/A		—		—		
	Other	339		32 (9.4)		1.37 (0.92-2.04)		.12		—		—		
**Education**														
	Primary and below	797		63 (7.9)		1.00		N/A		—		—		
	Junior high school	783		57 (7.3)		0.92 (0.631.33)		.64		—		—		
	Senior high school	360		21 (5.8)		0.72 (0.43-1.20)		.21		—		—		
	College	607		49 (8.1)		1.02 (0.69-1.51)		.91		—		—		
	Missing	21		0 (0)		—		—		—		—		
**Marital status^c^**														
	Single	950		84 (8.8)		1.00		N/A		—		—		
	Married or cohabiting	1144		72 (6.3)		0.69 (0.50-0.96)		.03		—		—		
	Divorced or widowed	457		34 (7.4)		0.83 (0.55-1.26)		.38		—		—		
	Missing	17		0 (0)		—		.98		—		—		
**Occupation**														
	Farmer	924		58 (6.3)		1.00		N/A		—		—		
	Other	1644		132 (8)		0.27 (0.16-2.64)		.10		—		—		
**Route of infection^c^**														
	Heterosexual intercourse	1480		102 (6.9)		1.00		N/A		—		—		
	Homosexual intercourse	951		72 (7.6)		1.11 (0.81-1.51)		.53		—		—		
	Intravenous drug use	21		6 (28.6)		5.41 (2.05-14.23)		<.001		—		—		
	Other	116		10 (8.6)		1.28 (0.65-2.51)		.48		—		—		
**Prior ARV^d^ exposure^c,e^**														
	No	2487		163 (6.6)		1.00		N/A		1.00		N/A		
	Yes	81		27 (33.3)		7.13 (4.37-11.62)		<.001		7.45 (4.50-12.34)		.001		
**CD4 cell count before ART (cell/mm^3^)**														
	0-199	962		65 (6.8)		1.00		N/A		—		—		
	≥200	1554		122 (7.9)		1.18 (0.86-1.61)		.31		—		—		
	Missing	52		3 (5.8)		0.85 (0.26-2.79)		.78		—		—		
**HIV subtype^c,e^**														
	CRF07_BC	1137		69 (6.1)		1.00		N/A		—		N/A		
	CRF08_BC	376		31 (8.2)		1.39 (0.89-2.16)		.14		1.09 (0.68-1.73)		.73		
	CRF01_AE	786		60 (7.6)		1.28 (0.89-1.83)		.18		1.27 (0.89-1.83)		.19		
	CRF55_01B	105		14 (13.3)		2.38 (1.29-4.39)		.006		2.61 (1.41-4.83)		.002		
	B	77		8 (10.4)		1.79 (0.83-3.88)		.14		1.61 (0.73-3.57)		.24		
	Other	87		8 (9.2)		1.57 (0.73-3.38)		.25		1.43 (0.65-3.14)		.37		
**Provincial-level administrative divisions^c^**														
	Sichuan	350		17 (4.9)		1.00		N/A		—		—		
	Hebei	175		13 (7.4)		1.57 (0.75-3.32)		.24		—		—		
	Jilin	206		19 (9.2)		1.99 (1.01-3.92)		.047		—		—		
	Jiangsu	335		24 (7.2)		1.51 (0.80-2.87)		.21		—		—		
	Zhejiang	385		34 (8.8)		1.90 (1.04-3.46)		.04		—		—		
	Hubei	362		20 (5.5)		1.15 (0.59-2.23)		.69		—		—		
	Chongqing	378		20 (5.3)		1.09 (0.56-2.13)		.79		—		—		
	Yunnan	377		43 (11.4)		2.52 (1.41-4.51)		.002		—		—		

^a^N/A: not applicable.

^b^Not available.

^c^Variables screened by stepwise regression.

^d^ARV: antiretroviral drugs.

^e^Variables selected into multivariable logistic regression models (without prior ARV exposure serving as the reference group and with HIV subtype represented as 5 dummy variables with CRF07_BC serving as the reference group).

### Molecular Transmission Network

**Figure 1:**
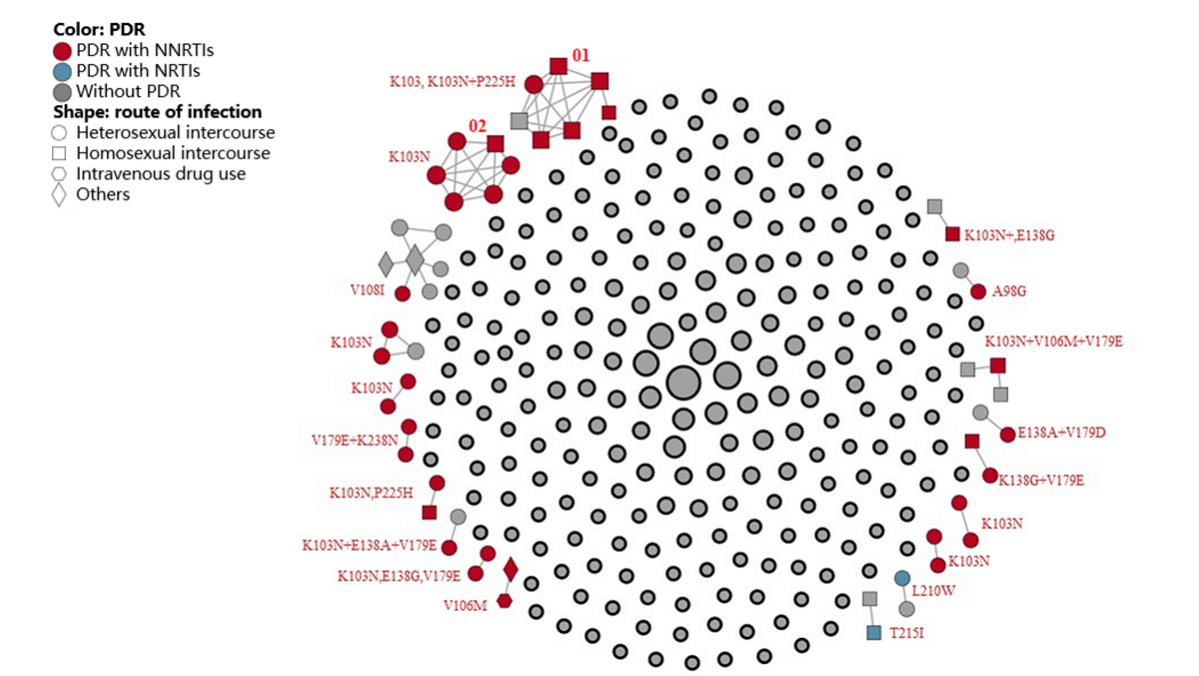
Molecular transmission network among HIV-positive individuals in 2022 in China.

Of the 2568 sequences in the PR/RT regions, 13 sequences were removed because the sequences in the PR/RT regions were shorter than 1000 bp or the number of mixed bases was >5%. Hence, a total of 2555 sequences in the PR/RT regions were used to construct a molecular transmission network. Under the optimal genetic distance threshold of 0.01 substitutions per site, 618 (24.2%) sequences (nodes) were linked to a total of 253 clusters (size range 2-13). We identified 38 sequences with PDR strains among 19 clusters. Most PDR strains linked to clusters had resistance to NNRTIs (36 of 38 sequences); DRM sites included K103, E138, V179, P225, V106, V108, L210, T215, P225, K238, and A98. Individual PDR status was not statistically significantly associated with belonging to a cluster. Cluster 01 with PDR consisted of 6 nodes in Jiangsu. Cluster 02 included HIV-positive individuals from 5 nodes in the same clinic sites in Zhejiang and 1 node in Hubei (Figure 1).

## Discussion

In 2022, a cross-sectional survey was conducted to examine the prevalence of PDR among HIV-positive individuals in 8 PLADs in China. The results showed that the overall prevalence of PDR was 7.4%. According to the WHO’s qualitative classification of PDR prevalence levels, a prevalence below 5% is considered low, a prevalence between 5% and 15% is moderate, and a prevalence ≥15% is high [5]. Therefore, the overall prevalence of PDR in 2022 in China and those of 7 PLADs (all but Sichuan) were considered moderate. The prevalence of PDR in 13 PLADs and 31 PLADs in China was 6.8% and 4.4% in 2017 and 2018, respectively [34,35]. Monitoring and surveillance are needed to assess the extent to which resistant strains have spread, and new technologies and strategies should be applied to effectively curb the spread of PDR.

Our study revealed that the PDR rate of NNRTIs was 6.3%. However, the PDR NNRTI prevalence was 10.3% for Yunnan. We observed higher PDR rates in Zhejiang and Yunnan in 2022 compared to surveys conducted in 2018 (3.7% in Zhejiang and 3.8% in Yunnan) and this increase in PDR prevalence was driven mainly by NNRTI resistance. According to WHO ART guidelines developed in 2016, it is recommended to consider implementing supplementary first-line antiviral treatment plans when the prevalence of PDR is equal to or greater than 10% for NNRTIs in HIV-positive individuals who are starting first-line ART [36]. Further research is needed to identify the underlying reasons for the high NNRTI PDR rates in Yunnan. From 2014 to 2020, 21 of 30 countries that reported investigations to the WHO had an NVP or EFV PDR rate that exceeded 10% among HIV-positive individuals starting first-line ART [37]. In our study, we found that the prevalence of EFV and NVP DR was 6.1% and 6.3%, respectively. The main DRM sites identified were K103, E138, V179, and V106. These results are consistent with earlier surveys conducted in China in 2015 and 2018 [32,34]. Studies from Zimbabwe, Kenya, and Argentina have identified K103 as the most common DRM site for NNRTIs used as first-line antiviral drugs [38-40]. National HIV treatment strategies and regimens should be reconsidered given the high prevalence of NNRTI DR. In 2023, the *National Free HIV Antiviral Treatment Drug Treatment Manual* was released in China, and rilpivirine was added as the first-line treatment regimen [41]. The RPV resistance rate in our study was 5.3% (137/2568). The PDR after rilpivirine is applied to first-line treatment alternatives should be continuously monitored.

Our 2022 study indicated that PDR rates have increased in China over the past 7 years for NRTI DR (from 1.1% in 2015 and 0.8% in 2018 to 1.2% in 2022) and PI DR (from 0.02% in 2015 and 0.07% in 2018 to 0.2% in 2022) [34]. Nonetheless, PDR rates for NRTIs and PIs in China generally appear to be lower than the WHO’s estimated regional PDR rates in Africa (NRTI PDR: 6.1%; PI PDR: 0.3%), the Americas (NRTI PDR: 6.4%; PI PDR: 0.8%), Southeast Asia (NRTI PDR: 3.1%; PI PDR: 0.4%), and the Western Pacific (NRTI PDR: 4%; PI PDR: 0%) [37].

Results of the multivariable analysis indicated that individuals who had prior ARV drug exposure had 7.45 times greater odds of PDR compared to those without prior ARV drug exposure. Such findings are consistent with research from Mexico, African countries, and Northeast China [42-44]. There was an increasing trend of the proportion of CRF55_01B samples, rising from 2.3% in 2015 to 3.9% in 2018 and 4.1% in 2022 in China [34]. Additionally, participants with the CRF55_01B subtype had 2.61 times greater odds of PDR (OR 2.61, 95% CI 1.41-4.83) compared to participants whose subtype was CRF07_BC. This may be due to association with V179 as a characteristic DR site of CRF55_01B, and V179 being frequently associated with E138G and causing low resistance to EFV and NVP [45]. Controlling the spread of CRF55_01B may be helpful for mitigating the proliferation of drug resistance. Furthermore, it is imperative to enhance the quality and effectiveness of intervention services for HIV-positive individuals.

In this study, we did not detect any statistically significant associations between having a PDR strain and belonging to clusters. Such results are similar with findings from Shijiazhuang city, Hebei Province and Liangshan region, Sichuan Province [46,47]. One possible explanation for these findings is that the transmission potential of DR strains is weaker than that of nonresistant wild strains [47,48]. The predominant subtype among the sequences in the molecular transmission network was CRF07_BC (representing 46.6% of strains), which is consistent with findings from a 2018 national molecular transmission network survey in China [32]. Clustered transmission of K103N DRMs in our study was observed in the molecular transmission network and is similar to findings from molecular transmission network studies in coastal Kenya [49]. It is worth noting that 6 DR strains in cluster 01 were from HIV-positive individuals at identical sites in Jiangsu Province. Epidemiological data indicated that all 6 were adult men aged younger than 30 years, of whom 5 reported homosexual transmission and 1 reported heterosexual transmission. Cluster 02 contained 6 samples with DR strains from HIV-positive individuals in the same clinic sites in Zhejiang Province, of whom 5 reported heterosexual transmission and 1 reported homosexual transmission, suggesting a possible clustered transmission of DR strains in that area.

Our study has several limitations. Due to sampling limitations, clinic sites were established in different counties and districts, and only 20-80 patients were enrolled in each site during a certain period of time. This may have resulted in smaller observable clusters (containing 2-13 nodes) in the molecular network and a lower network access rate. In addition, when detecting drug-resistant strains using genotyping, Sanger sequencing may not be able to detect up to 20% of less prevalent PDR strains [24]. This may have resulted in an underestimation of the PDR rate in HIV-positive individuals.

The findings from our 2022 PDR survey in China indicate a need to review and reconsider the standard ART protocol in some PLADs. Additional resources and strategies are needed in regions within China experiencing a higher prevalence of PDR. Possible interventions include extending pilot genotypic PDR testing to rapidly adjust medication regimens for HIV-positive individuals identified with PDR and targeted measures to improve medication compliance and prevention.

## Appendix

Multimedia Appendix 1 Sample size of HIV-positive individuals in 8 provincial-level administrative divisions in 2022 in China.

## Abbreviations

ART: antiretroviral therapy
ARV: antiretroviral drugs
DR: drug resistance
DRM: drug resistance mutation
EFV: efavirenz
NNRTIs: nonnucleoside reverse transcriptase inhibitors
NRTIs: nucleoside reverse transcriptase inhibitors
NVP: nevirapine
OR: odds ratio
PDR: pretreatment HIV drug resistance
PIs: protease inhibitors
PLADs: provincial-level administrative divisions
PR: protease
RT: reverse transcriptase
WHO: World Health Organization
