# Supplementary material for: Pretreatment HIV Drug Resistance and the Molecular Transmission Network Among HIV-Positive Individuals in China in 2022: Multicenter Observational Study
Source: JMIR Public Health Surveill. 2023 Nov 17;9:e50894. doi: 10.2196/50894 (PMC10692882; doi:10.2196/50894)
Supplement: Multimedia Appendix 1 [file publichealth_v9i1e50894_app1.docx]

Multimedia Appendix 1. Sample size of HIV-positive individuals in eight provincial-level administrative divisions in 2022 in China

| Areas | Total number of clinics | Number of  Patients estimated who initiated ART  during 6-month  survey period in 2022 | Number Of Clinics included in study | Minimum necessary sample size (individuals) | Total number of ART initiators  included in sampling | Total of  sampled  patients | Total of  eligible  patients |
| --- | --- | --- | --- | --- | --- | --- | --- |
| Total | 748 | 25021 | 132 | 1298 | 3373 | 2869 | 2844 |
| Hebei | 23 | 1055 | 11 | 179 | 400 | 218 | 213 |
| Jilin | 55 | 598 | 13 | 171 | 433 | 228 | 228 |
| Jiangsu | 54 | 2205 | 16 | 158 | 400 | 375 | 370 |
| Zhejiang | 96 | 2125 | 18 | 158 | 540 | 418 | 414 |
| Hubei | 94 | 1464 | 16 | 158 | 400 | 418 | 418 |
| Chongqing | 77 | 3553 | 18 | 158 | 400 | 410 | 407 |
| Sichuan | 173 | 9299 | 20 | 158 | 400 | 400 | 396 |
| Yunnan | 176 | 4722 | 20 | 158 | 400 | 402 | 398 |
